# Molecules, meaning, and physiology: disentangling the active ingredients of psychedelic therapeutics

**DOI:** 10.3389/fnins.2026.1892474

**Published:** 2026-08-03

**Authors:** Kim P. C. Kuypers

**Affiliations:** Department of Neuropsychology & Psychopharmacology, Faculty of Psychology & Neuroscience, Maastricht University, Maastricht, Netherlands

**Keywords:** autonomic physiology, breathwork, experiential, neurochemical, psychedelics, vagus nerve stimulation

## Abstract

Psychedelic-assisted therapy has shown rapid and durable benefits across multiple psychiatric conditions, yet the mechanisms underlying these effects remain incompletely understood. Full-dose psychedelic sessions simultaneously engage serotonergic neurochemistry, high-intensity subjective experience, and autonomic-interoceptive physiology, making it difficult to determine which components are necessary or sufficient for therapeutic change. This perspective proposes that three partially separable research approaches, a single low dose of a psychedelic, high-ventilation breathwork, and vagus nerve stimulation (VNS), offer complementary mechanistic probes for disentangling these pathways. A single, sub-psychedelic dose provides a model of low-intensity 5-HT2A engagement that isolates pharmacological and early plasticity-related effects while minimizing experiential and autonomic confounds. Breathwork induces robust experiential and autonomic shifts through CO_2_-driven physiological perturbation and interoceptive amplification, enabling investigation of experiential and autonomic contributions in the absence of serotonergic pharmacology. VNS, in turn, modulates vagal-autonomic and interoceptive circuits without inducing altered states of consciousness, offering a controlled method for probing autonomic influences on affective and cognitive processes. Examining these perturbations within shared experimental frameworks may clarify how neurochemical plasticity, experiential meaning-making, and autonomic recalibration interact to support psychological change. This mechanistic precision can guide the development of interventions that are safer, more scalable, and more individually tailored than current full-dose psychedelic protocols.

## Introduction

Mental health disorders represent a growing global crisis, with rising rates of depression, anxiety, and burnout despite widespread use of pharmacological and psychotherapeutic treatments (Rehm and Shield, 2019). Psychedelic-assisted therapy has emerged as a promising alternative, demonstrating rapid and durable improvements across conditions including major depressive disorder, post-traumatic stress disorder, addiction, and end-of-life anxiety (Mitchell and Anderson, 2024). Although these clinical findings have generated considerable enthusiasm, the mechanisms through which psychedelics exert their therapeutic effects remain incompletely understood.

Historically, psychiatry has alternated between biological models, which emphasize serotonergic dysfunction and neurochemical imbalance (Troisi, 2022), and experiential models, which highlight meaning-making, emotional breakthrough, and shifts in self-related processing (Ungar-Sargon, 2025). Psychedelics complicate this dichotomy because they simultaneously alter neurochemistry, conscious experience, and autonomic-interoceptive physiology (Nichols et al., 2017; Vollenweider and Preller, 2020). When multiple pathways are perturbed at once, it becomes difficult to determine which are necessary or sufficient for therapeutic change, and whether improvements arise from receptor-level plasticity, the quality of the acute experience, autonomic recalibration, or interactions among these domains.

The central aim of this article is to clarify how these mechanistic pathways contribute to therapeutic outcomes by examining three research lines that perturb them in partially separable ways, i.e., low-dose psychedelics, breathwork-induced altered states, and vagus nerve stimulation (VNS). Rather than focusing on microdosing, which involves repeated administration, this framework emphasizes a single, low, sub-psychedelic dose that allows researchers to isolate acute pharmacological effects with minimal experiential confounds. By comparing these three approaches, it becomes possible to investigate how serotonergic modulation, experiential intensity, and autonomic-interoceptive dynamics each contribute to psychological change, and to use low-dose psychedelics, breathwork, and VNS as complementary mechanistic probes to disentangle these pathways.

## Candidate mechanisms of therapeutic change

**Figure 1:**
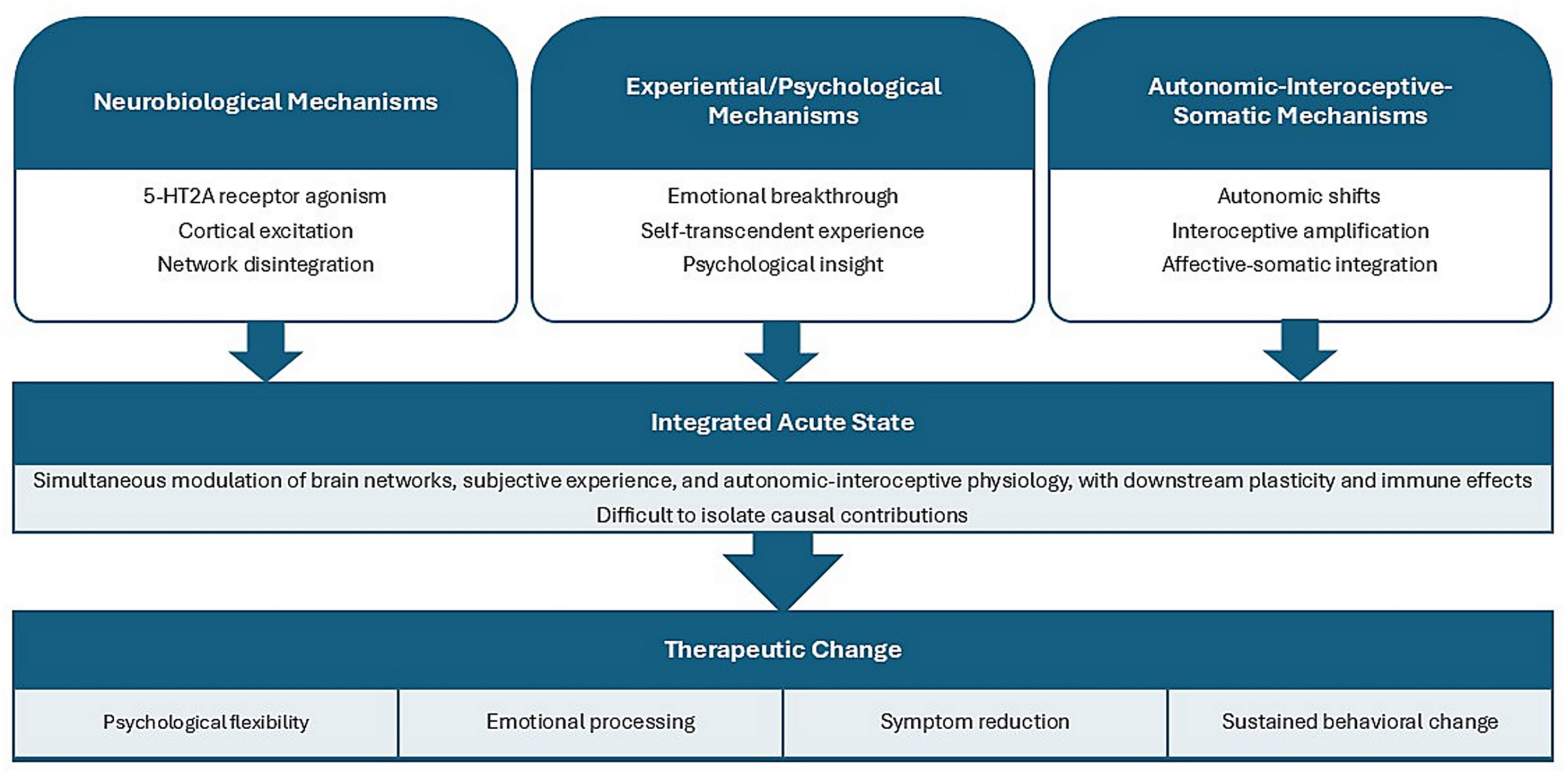
Three mechanistic pathways of psychedelic action. Full-dose psychedelics modulate all three domains simultaneously.

Psychedelics appear to exert their therapeutic effects through interacting processes spanning neurochemical, experiential, and autonomic-interoceptive domains. Although these pathways are often discussed separately, they unfold simultaneously during a full-dose psychedelic session, making it difficult to determine which are necessary or sufficient for clinical improvement. The following subsections outline the evidence supporting each mechanistic pathway and highlight the challenges associated with isolating their individual contributions (Figure 1).

### Molecular and network plasticity pathway

Classic psychedelics act primarily as 5-HT2A receptor agonists, initiating a cascade of neurobiological effects that reshape cortical and large-scale network dynamics. Activation of 5-HT2A receptors on deep-layer pyramidal neurons increases neuronal excitability and disrupts the stability of intrinsic networks, including the default mode, salience, and frontoparietal control networks (Vollenweider and Kometer, 2010; Banushi and Polito, 2023). These network-level changes are accompanied by increases in neural signal diversity and cognitive flexibility, which have been proposed as markers of a more entropic and less prediction-bound brain state (Schartner et al., 2017; Timmermann et al., 2019; Doss et al., 2021).

Preclinical work further demonstrates that psychedelics enhance synaptic and dendritic plasticity through coordinated activation of 5-HT2A, BDNF–TrkB, and mTOR signaling pathways. Stimulation of the 5-HT2A receptor is required for psychedelic induced neuritogenesis and spinogenesis, and these structural changes depend on downstream TrkB and mTOR activation (Ly et al., 2018). Systematic reviews similarly show that psychedelics increase BDNF expression and plasticity-related gene transcription in a 5-HT2A-dependent manner (De Vos et al., 2021). Recent mechanistic work further indicates that serotonergic psychedelics recruit an integrated 5-HT2A-TrkB signaling network, linking receptor activation to structural, transcriptional, and metabolic plasticity outputs (Virenque et al., 2026; Taddei-Tardón et al., 2026).

Together, these findings suggest that psychedelics transiently shift the brain into a more plastic and flexible state through 5-HT2A-dependent molecular and network-level mechanisms. However, enhanced plasticity is not unique to psychedelics. Compounds such as ketamine similarly increase synaptic and dendritic plasticity while producing a dissociative rather than psychedelic phenomenology (Abdallah et al., 2015; Duman et al., 2019). Non-hallucinogenic psychoplastogens likewise promote structural plasticity without altering consciousness (Olson, 2025), demonstrating that plasticity can be pharmacologically induced in the absence of psychedelic-type subjective effects. This dissociation between plasticity and phenomenology indicates that receptor-level and network-level plasticity alone are unlikely to fully account for therapeutic benefit, and that additional processes, such as autonomic, interoceptive, or experiential factors, may shape clinical outcomes.

### Experiential pathway

A second body of research emphasizes the contribution of the acute subjective experience. Multiple clinical trials have reported that the intensity of mystical-type experiences, emotional breakthrough, and psychological insight during psychedelic sessions predicts the magnitude of subsequent symptom reduction across conditions including depression, anxiety, addiction, and cancer-related distress (Roseman et al., 2018; Yaden and Griffiths, 2020; Ko et al., 2022; Romeo et al., 2025). Meta-analytic and scoping reviews similarly report consistent associations between experiential intensity and therapeutic outcomes (Ko et al., 2022; Deutsch et al., 2026). These findings have led some authors to propose that the subjective experience may play a mechanistic role in driving psychological change (Yaden and Griffiths, 2020), potentially by facilitating emotional processing, autobiographical reappraisal, or shifts in self-related beliefs (Kangaslampi, 2023; Kangaslampi and Lietz, 2025). At the same time, the causal status of these experiences remains debated. Experiential intensity may reflect downstream consequences of receptor-level engagement rather than a distinct mechanism, and not all individuals who undergo profound or high-intensity psychedelic experiences show clinical improvement, indicating that strong phenomenology alone is insufficient to account for therapeutic outcomes (Olson, 2020).

### Autonomic-interoceptive pathway

A third mechanistic pathway involves autonomic and interoceptive physiology. Psychedelics reliably modulate autonomic balance, with studies reporting dose-dependent increases in heart rate, blood pressure, and sympathetic arousal (Holze et al., 2022). Autonomic activation correlates with the emotional or perceptual intensity of the experience, suggesting a close coupling between physiological arousal and experiential depth (Rosas et al., 2023). DMT-induced peak states show a characteristic pattern of sympatho-vagal coactivation, and pre-dose autonomic balance predicts the depth and emotional tone of the ensuing experience (Bonnelle et al., 2024). These findings align with broader evidence linking vagal tone, CO_2_ sensitivity, and interoceptive accuracy to emotional regulation and mental health outcomes (Critchley and Garfinkel, 2017; Khalsa et al., 2018). Psychedelic states also involve pronounced somatic sensations, shifts in bodily awareness, and alterations in interoceptive precision (Studerus et al., 2010; Letheby and Gerrans, 2017), suggesting that changes in autonomic-interoceptive dynamics may contribute meaningfully to therapeutic processes. Yet, as with the experiential pathway, autonomic changes occur concurrently with neurochemical and phenomenological alterations, making it difficult to determine their independent contribution.

Together, these three pathways illustrate the complexity of psychedelic mechanisms. Full-dose psychedelic sessions perturb neurochemical, experiential, and autonomic systems simultaneously, and these domains interact in ways that are not yet fully understood. Disentangling their relative contributions requires experimental approaches that selectively engage one pathway while minimizing effects on the others. The subsequent sections describe how a single low dose of psychedelics, breathwork-induced altered states, and VNS can serve as complementary probes to isolate these mechanisms.

## A low dose of a psychedelic as a probe of low-intensity pharmacology

Administering a single low dose of a psychedelic (a “minidose”), such as 20 μg LSD or 5 mg psilocybin (Nutt et al., 2025), provides a means of examining serotonergic pharmacology in the relative absence of intense subjective effects (Hutten et al., 2020a). This approach is conceptually distinct from microdosing, which involves repeated administration over days or weeks (Kuypers et al., 2019). A single low dose enables researchers to isolate acute receptor-level and network-level effects without the cumulative physiological uncertainties associated with multi-day dosing schedules, including the ongoing debate about the relevance of 5-HT2B-mediated valvulopathy risk at low or intermittent doses (Rouaud et al., 2024; Effinger et al., 2025).

Low-dose psychedelic studies have reported subtle but measurable effects on mood, attention, and affective processing. Placebo-controlled work has shown small improvements in mood and attentional performance following low-dose LSD (Hutten et al., 2020a), and alterations in time perception and emotional processing at doses below the threshold for full psychedelic phenomenology (Yanakieva et al., 2019; Molla et al., 2024; Glazer et al., 2023). Neuroimaging and pharmacokinetic studies demonstrate that even a low dose of psilocybin (3 mg) produces detectable 5-HT2A receptor occupancy, confirming that a low dose engages serotonergic targets despite minimal subjective effects (Madsen et al., 2019). Complementary physiological and neural findings indicate that a single low dose can increase serum BDNF (Hutten et al., 2020b), alter resting-state functional connectivity (Bershad et al., 2020), and modulate EEG signatures associated with cortical excitation (Hutten et al., 2024). These results suggest that low-dose psychedelics produce biologically meaningful effects on receptor-level signalling and network dynamics.

At the same time, the literature on the acute effects of low-dose psychedelics remains mixed. Although small but reliable improvements in mood are often observed, several placebo-controlled studies have failed to detect robust or consistent changes in cognitive performance (Totomanova et al., 2025). Acute low-dose studies in both healthy volunteers and individuals with low mood further show that subjective responses at these doses are shaped by individual differences (Hutten et al., 2020a; Hutten et al., 2024; Molla et al., 2024), and expectancy-related influences have also been shown to contribute substantially to acute effects (Szigeti and Heifets, 2024). Importantly, although expectancy (most likely) contributes to (subjective) responses, studies nonetheless demonstrate clear acute pharmacological engagement (Hutten et al., 2020b; Hutten et al., 2024; Bershad et al., 2020; Cavanna et al., 2022; Madsen et al., 2019); yet placebo-controlled evaluations of acute therapeutic effects of single low doses are largely absent (e.g., Mueller et al., 2025). Consequently, the lack of demonstrated clinical benefit reflects limited systematic investigation rather than evidence that low-intensity serotonergic modulation is ineffective.

Single low doses of psychedelics thus provide a useful probe for isolating serotonergic pharmacological engagement, producing subtle 5-HT2A-mediated neural and physiological effects without intense phenomenology.

## Breathwork as a probe of experience and autonomic physiology

High-arousal breathwork modalities, including holotropic and shamanic practices, reliably induce altered states of consciousness in the absence of serotonergic pharmacology (Fincham et al., 2023a,b; Havenith et al., 2025). These practices have been shown to evoke experiential states that share phenomenological features with psychedelic sessions, including perceptual alterations, emotional release, autobiographical imagery, and shifts in self-related processing (Havenith et al., 2025). As such, breathwork provides a means of examining the experiential and autonomic components of psychedelic-like states while avoiding direct engagement of 5-HT2A receptors.

The physiological mechanisms underlying breathing are well characterized. Rapid, deep breathing produces a marked reduction in arterial CO_2_ (hypocapnia), leading to respiratory alkalosis and consequent cerebral vasoconstriction (Ainslie and Duffin, 2009). These changes alter sensory processing and can contribute to visual distortions, paraesthesia, and shifts in bodily awareness (Lum, 1975; Gardner, 1996). Hypocapnia also increases neuronal excitability and amplifies interoceptive signals, producing heightened awareness of cardiac, respiratory, and visceral sensations (Meuret et al., 2008). Sympathetic activation during the hyperventilation phase (Gardner, 1996) is often followed by a parasympathetic rebound during breath retention or recovery, creating a dynamic autonomic profile consistent with respiratory-autonomic coupling (Jerath et al., 2006; Guyenet, 2014) and mirroring the oscillatory autonomic patterns observed during psychedelic experiences (Bonnelle et al., 2024). These physiological shifts are closely linked to emotional lability and somatic release (Fincham et al., 2023a,b), as well as the emergence of autobiographical material (Rock et al., 2015), suggesting that autonomic perturbation may play a central role in shaping the experiential depth of breathwork-induced states.

Naturalistic and experimental studies support the relevance of these mechanisms for understanding psychedelic-like effects. In a naturalistic comparison, emotional and cognitive changes reported after breathwork sessions were comparable in magnitude to those observed following ayahuasca ceremonies, despite the absence of a psychoactive compound (Khubsing et al., 2024; Rossi et al., 2026). Havenith et al. (2025) similarly demonstrated that breathwork can elicit experiential and emotional effects resembling those produced by serotonergic psychedelics (Havenith et al., 2025). These findings align with broader evidence linking autonomic and interoceptive processes to emotional regulation, affective updating, and therapeutic change (Khalsa et al., 2018; Owens et al., 2018). Breathwork therefore provides a mechanistic probe for isolating the contributions of experiential intensity and autonomic-interoceptive dynamics to psychological outcomes.

At the same time, breathwork-induced states differ from psychedelic states in important ways. The phenomenology of breathwork is shaped by CO_2_-driven physiological perturbation rather than receptor-level modulation, and the temporal dynamics of autonomic activation differ from those observed during serotonergic psychedelic sessions. Research on high-ventilation breathwork remains dominated by naturalistic workshop settings with limited experimental control (Rock et al., 2015), and the absence of standardized breathing protocols complicates comparisons across studies (Fincham et al., 2023a,b; Banushi et al., 2023). These limitations underscore the need for controlled, paced-breathing paradigms with continuous physiological monitoring to clarify how autonomic and interoceptive processes contribute to the emergence of psychedelic-like states.

Breathwork thus can serve as a complementary probe by inducing intense experiential and autonomic shifts without serotonergic pharmacology, allowing researchers to isolate experiential-autonomic contributions to psychological change.

## Vagus nerve stimulation as a probe of autonomic physiology

Vagus nerve stimulation provides a mechanistically precise method for probing autonomic physiology because it directly modulates parasympathetic efferents, afferent interoceptive pathways, and central autonomic networks (Frangos et al., 2015). Both invasive and transcutaneous approaches reliably increase parasympathetic activity, enhance heart-rate variability, and shift sympatho-vagal balance toward greater vagal dominance (Clancy et al., 2014; Badran et al., 2018). Neuroimaging studies demonstrate that VNS engages the nucleus tractus solitarius, insula, amygdala, and prefrontal regions (Frangos et al., 2015; Badran et al., 2018), indicating that stimulation influences circuits involved in interoception, emotional regulation, and bodily self-processing (Berntson and Khalsa, 2021; Ben Shalom, 2022; Fermin et al., 2023). These findings align with broader evidence showing that vagal afferents play a central role in shaping affective and cognitive states (Bonaz et al., 2017; Breit et al., 2018).

A key advantage of VNS as an experimental probe is the ability to control stimulation parameters with high precision. Frequency, pulse width, duty cycle, and electrode placement each influence the balance of afferent and efferent fiber recruitment, and these parameters determine the magnitude and direction of autonomic effects (Yakunina et al., 2017; Thompson et al., 2021). Frequencies in the 20–30 Hz range preferentially recruit peripheral myelinated A-fibers, producing robust activation of nucleus tractus solitarius (NTS)-locus coeruleus-insula pathways (Yakunina et al., 2017; Badran et al., 2018; Ahmed et al., 2022). Although VNS does not engage cortical 5-HT2A receptors, selectively activating peripheral vagal afferents can modulate affective and interoceptive processing by influencing serotonergic function indirectly through NTS projections to the dorsal raphe nucleus, thereby altering mood-related circuits independently of 5-HT2A signaling (Dorr and Debonnel, 2006). Pulse width, current amplitude, and respiratory-phase timing further shape the depth and efficacy of vagal fiber recruitment (Sclocco et al., 2020), and these parameter-dependent effects interact with individual differences in, for example, auricular innervation, baseline vagal tone, and interoceptive sensitivity (Farmer et al., 2021). Clinical studies likewise show that optimal stimulation parameters vary across patients and require iterative adjustment (Groves and Brown, 2005), underscoring that VNS is a heterogeneous, state-dependent neuromodulation tool that cannot be assumed to exert uniform effects across individuals.

VNS thus serves as a targeted probe of autonomic mechanisms, modulating vagal signaling without serotonergic or experiential confounds and complementing the distinct perturbations produced by low-dose psychedelics and breathwork.

## Toward an integrated mechanistic framework

Because full-dose psychedelic sessions conflate serotonergic, experiential, and autonomic effects, rendering them mechanistically opaque, low-dose psychedelics, breathwork, and VNS provide complementary probes that perturb these domains more selectively and thereby clarify their respective contributions to therapeutic change.

**Figure 2:**
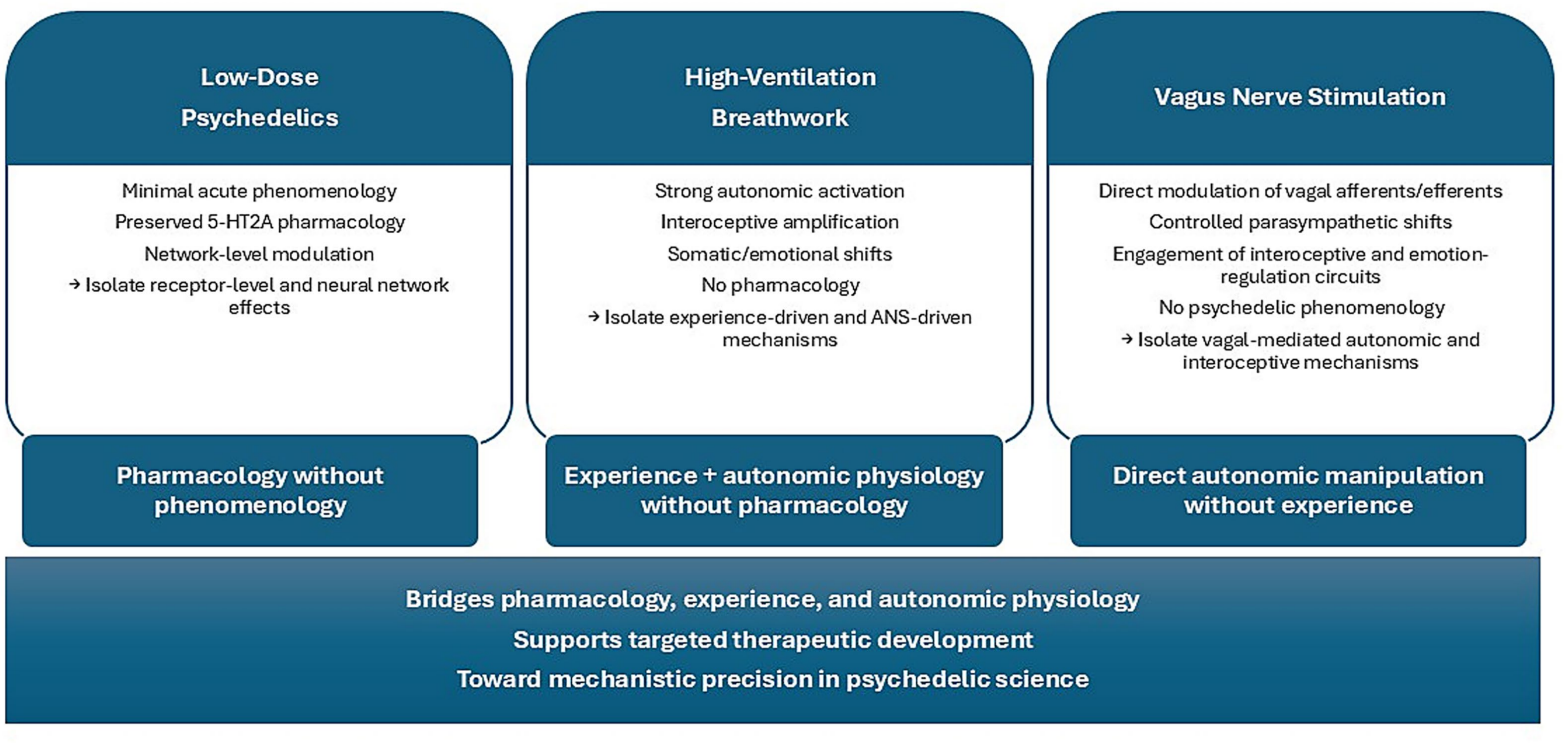
Integrating mechanistic pathways of psychedelic action with experimental probes of pharmacology, experience, and autonomic physiology. ANS, Autonomic Nervous System.

A single low dose of a psychedelic engages 5-HT2A receptors and produces subtle shifts in plasticity and network dynamics without the intense phenomenology of full-dose sessions (Hutten et al., 2020a; Madsen et al., 2019), allowing serotonergic contributions to be examined with minimal experiential or autonomic confounds. Breathwork, by contrast, elicits strong experiential states and pronounced autonomic shifts through CO_2_-driven physiological perturbation and interoceptive amplification (Fincham et al., 2023a,b; Havenith et al., 2025). VNS selectively modulates autonomic pathways and interoceptive circuits (Clancy et al., 2014; Frangos et al., 2015), offering a controlled means of probing how vagal signaling shapes emotional regulation and bodily awareness. Together, these approaches suggest that therapeutic change might emerge from interactions between neurochemical plasticity, experiential meaning-making, and autonomic recalibration, with autonomic state influencing experiential depth (Bonnelle et al., 2024) and experiential intensity interacting with neurobiological plasticity mechanisms to influence the durability of therapeutic effects (Yaden and Griffiths, 2020) (Figure 2).

**Figure 3:**
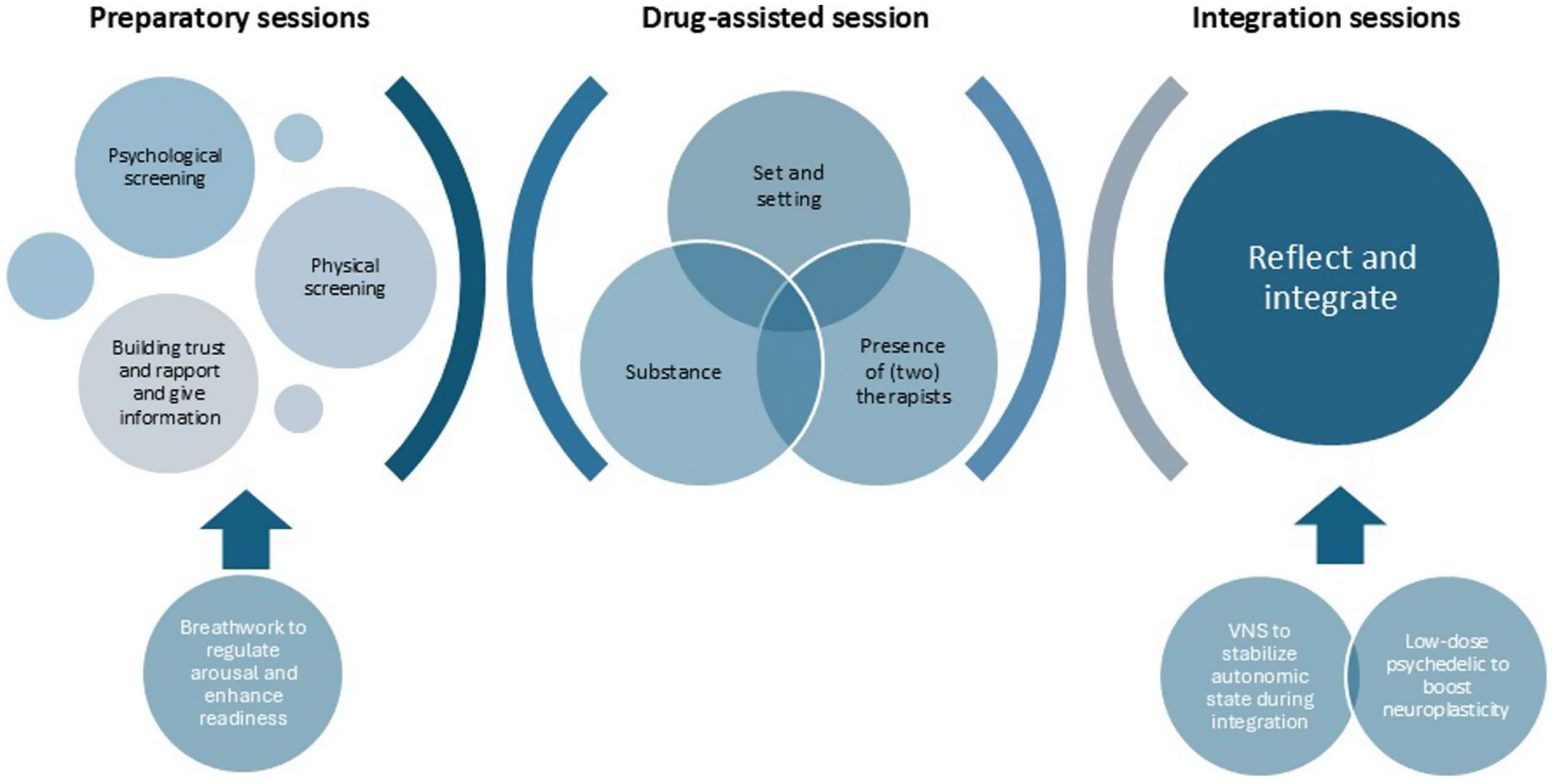
Conceptual illustration of how mechanistic insights from low-dose psychedelics, breathwork, and VNS can inform the development of novel, sequenced interventions designed to maximize therapeutic benefit while minimizing risk.

Taken together, this framework highlights how selectively perturbing pharmacological, experiential, and autonomic pathways can generate mechanistic insights that inform the design of combined or sequenced interventions, for example, using breathwork to regulate arousal and enhance psychological readiness before a psychedelic session (Rosenbaum et al., 2024), or applying VNS during integration to stabilize autonomic state and support the consolidation of experiential and plasticity-related changes, while low‑dose psychedelics can be used in distinct sessions to enhance neuroplasticity, with the possibility of sequencing or combining these approaches in future work (Figure 3).

## Future directions

Future research should prioritize mechanistically informed, multimodal study designs capable of testing the framework outlined above. For low-dose psychedelics, adequately powered, placebo-controlled crossover trials are needed, incorporating receptor-level measures such as 5-HT2A occupancy (Madsen et al., 2019). Although peripheral 5-HT2A expression has not yet been systematically examined in psychedelic studies, platelets do express 5-HT2A receptors, and prior work in depressed patients (Amidfar et al., 2017) suggests that peripheral assays may offer a feasible avenue for probing serotonergic biology in future mechanistic studies. Rigorous expectancy-control procedures will also be essential; Broadened drug-class instructions, as used in recent laboratory studies (Molla et al., 2024), may reduce condition-guessing and help address the substantial expectancy effects observed in low-dose research (Szigeti and Heifets, 2024). Dose–response designs are also needed, as small variations in low-dose ranges can produce nonlinear changes in subjective and cognitive outcomes (Hutten et al., 2020a; Holze et al., 2021b). Comparative within-subject studies evaluating different psychedelics, for example, psilocybin versus LSD, provide a powerful approach for isolating compound-specific versus shared serotonergic mechanisms (Holze et al., 2022). Active comparators may also be valuable in low-dose studies, where the goal is to match subtle psychoactive and autonomic effects rather than full psychedelic phenomenology. Comparator choice should be linked to the primary endpoint, for example, mild stimulants for attentional outcomes or SSRIs for affective endpoints. While the use of active comparators in full psychedelic doses has failed to preserve blinding (Belinger et al., 2026), it may still function as credible low-intensity controls when perceptual effects are minimal. Expectancy-management strategies referenced above further support blinding integrity in these designs.

Breathwork research would benefit from greater experimental control, as existing studies rely heavily on protocols with heterogeneous pacing, depth, and instruction, a limitation highlighted in recent systematic reviews (Zaccaro et al., 2018; Fincham et al., 2023a,b; Banushi et al., 2023). Recent methodological work outlining a comparative breathing-technique protocol (Cavarra et al., 2025; Cavarra and Kuypers, 2025) illustrates the recognition that different breathing styles likely produce distinct autonomic and experiential profiles, yet systematic empirical comparisons remain scarce (e.g., Fincham et al., 2023a; Havenith et al., 2025). Standardized, experimentally paced breathing paradigms with continuous physiological monitoring could clarify how CO_2_ dynamics, sympathetic activation, and interoceptive amplification contribute to the emergence of psychedelic-like states (Fincham et al., 2023a,b; Havenith et al., 2025). Integrating measures of chemosensory sensitivity (e.g., CO_2_ responsivity), vagal tone, and interoceptive accuracy may help identify autonomic-interoceptive profiles that shape experiential depth and emotional release. Chemosensory and autonomic afferents are core components of the interoceptive system (Khalsa et al., 2018; Owens et al., 2018), making these measures well-suited for mechanistic comparisons across breathing techniques and serotonergic psychedelic states.

Neuromodulation approaches such as transcutaneous VNS (tVNS) similarly require systematic parameter mapping, including stimulation frequency, pulse width, duty cycle, and respiratory phase each influence afferent recruitment, yet few studies vary these parameters or examine their interaction with baseline autonomic state (Clancy et al., 2014; Badran et al., 2018; Farmer et al., 2021). Combining VNS with neuroimaging, autonomic monitoring, and computational modeling may help identify stimulation profiles that most effectively modulate interoceptive circuits (Yakunina et al., 2017). Incorporating individual differences in auricular innervation, baseline vagal tone, and interoceptive/autonomic sensitivity, factors known to shape variability in tVNS engagement (Groves and Brown, 2005; Yakunina et al., 2017), will be essential for understanding heterogeneous responsiveness. Future work should also distinguish between acute effects, reflecting immediate autonomic and interoceptive modulation, and effects emerging after repeated administration, which may involve cumulative changes in plasticity-related pathways, inflammatory signaling, and affective regulation (Bonaz et al., 2016; Thompson et al., 2021; Olsen et al., 2021).

Although fMRI and EEG remain central tools for characterizing neural and autonomic-interoceptive dynamics, fNIRS offers a valuable complementary modality for studies involving movement, breathwork, or neuromodulation (Pinti et al., 2020). Its tolerance for head motion and suitability for naturalistic or ambulatory settings make it well suited for tracking cortical hemodynamics during paced breathing, CO_2_-modulated states, or tVNS stimulation (Scholkmann et al., 2014). Recent methodological work further shows that fNIRS can reliably capture task-evoked hemodynamic responses in contexts where fMRI is impractical, including respiratory and autonomic challenges (Klein et al., 2024). Integrating fNIRS with EEG or autonomic monitoring provides a multimodal window into cortical-autonomic coupling, combining fast electrophysiological responses, slower hemodynamic changes, and real-time autonomic fluctuations under conditions where motion or stimulation parameters limit fMRI (Klein et al., 2024; Pinti et al., 2020; Scholkmann et al., 2014; Yang and Wang, 2025).

Heart-rate variability (HRV) is a promising transdiagnostic marker to include across these mechanistic probes, as it indexes dynamic autonomic regulation tightly linked to affective processing, emotion-regulation capacity, and mental-health outcomes (Kemp et al., 2010). Reduced resting HRV is consistently associated with depression, anxiety, and impaired emotion regulation, whereas higher HRV reflects greater regulatory capacity and resilience (Kemp et al., 2010). Classic psychedelics reliably modulate autonomic state, typically increasing heart rate and blood pressure during the acute experience (Holze et al., 2022), and recent work has shown that autonomic variability, including HRV-derived indices, fluctuates with experiential intensity (Bonnelle et al., 2024), suggesting that HRV may serve as a sensitive marker of autonomic-affective engagement in future studies. Fast or high-frequency breathing reduces HRV by suppressing respiratory sinus arrhythmia, as shorter respiratory cycles diminish vagally mediated respiratory-cardiac coupling (Yasuma and Hayano, 2004), and high-ventilation breathwork similarly induces strong sympathetic activation and CO_2_-driven physiological arousal that likely further reduces HRV during the practice, whereas VNS tends to increase HRV and vagal indices, reflecting enhanced parasympathetic engagement and autonomic flexibility (Clancy et al., 2014). Incorporating HRV into future multimodal designs would therefore allow researchers to quantify how these interventions differentially perturb autonomic control, link autonomic trajectories to changes in affective processing and symptomatology, and test whether shifts in HRV, acute or longitudinal, mediate improvements in psychological resilience and therapeutic outcome.

Future research should also place stronger emphasis on interindividual variability, as accumulating evidence shows that responses to psychedelics, breathwork, and VNS are shaped by a complex interplay of biological, psychological, and contextual factors (Studerus et al., 2012; Groves and Brown, 2005; Zaccaro et al., 2018). Variability in receptor binding and pharmacokinetics, including differences in 5-HT2A occupancy, metabolic phenotype, and CYP-mediated drug metabolism, may influence both acute phenomenology and longer-term outcomes (Madsen et al., 2019; Luethi et al., 2019). Age-related changes in neuroplasticity, autonomic flexibility, and serotonergic signaling further modulate responsivity (Meltzer et al., 1998; Burke and Barnes, 2006; Monahan, 2007), while sex-related hormonal dynamics introduce additional layers of variability through their effects on serotonergic and autonomic function (Barth et al., 2015; Usselman et al., 2016; Tanaka et al., 2003). Although empirical psychedelic studies have not yet focused specifically on women or menopausal status, recent perspective and review papers highlight the need to consider menstrual-cycle phase, estrogen fluctuations, and midlife hormonal transitions as potential modulators of affective and neurobiological responses (Shadani et al., 2024; Cohen and Blest-Hopley, 2025). Similar considerations apply to breathwork and VNS, as sex hormones and menopausal status influence autonomic regulation, CO_2_ chemosensitivity, and vagal reactivity, key pathways through which these interventions exert their effects (Tanaka et al., 2003; Slatkovska et al., 2006; Usselman et al., 2016). Psychological traits such as neuroticism, absorption, trauma history, and baseline symptom severity have been identified as predictors of both acute psychedelic experience and clinical outcome (Romeo et al., 2025; Haijen et al., 2018; Viljoen et al., 2025), and similar trait-level moderators are increasingly recognized as relevant for breathwork and VNS, given their influence on autonomic responsivity, interoceptive sensitivity, and stress-system reactivity (Thayer and Lane, 2000; Paulus and Stein, 2010; Zaccaro et al., 2018). These considerations underscore the need to examine heterogeneous samples spanning subclinical, clinical, and transdiagnostic populations. Frameworks such as RDoC provide a principled way to organize these individual differences across domains including negative valence, arousal regulation, cognitive control, and social processing (Kelly et al., 2021), and biology-informed precision-psychiatry frameworks emphasize the value of integrating neuroimaging, pharmacogenetics, and computational modeling to identify mechanism-based predictors of treatment response (Kas et al., 2025).

A further priority is to examine how immune, autonomic, and plasticity-related pathways interact to shape therapeutic outcomes across these mechanistic probes. Psychedelics modulate cytokine signaling and microglial activity through 5-HT2A-mediated mechanisms (Thuery et al., 2025), while breathwork and VNS influence inflammation via autonomic routes (Kox et al., 2014; Koopman et al., 2016), underscoring the need to integrate immune biomarkers into multimodal designs to clarify how inflammatory states interact with plasticity, interoception, and experiential processes. Although psychedelics reliably induce plasticity-related molecular changes in animal models (De Vos et al., 2021; Moliner et al., 2023), human evidence for peripheral BDNF changes remains inconsistent (Calder et al., 2025); VNS modulates neuromodulatory pathways that support cortical plasticity (Hays et al., 2013), and breathing practices influence autonomic, interoceptive, and BDNF-related mechanisms implicated in cognitive and affective regulation (Krishna et al., 2024). To compare these probes directly, future studies should combine physiological and molecular readouts with standardized experiential assessments such as the 5D-ASC (Schmidt and Berkemeyer, 2018), enabling alignment across interventions that differ in phenomenology and autonomic impact. Comparative within-subject designs, testing low-dose psychedelics, breathwork, and VNS in the same participants, would allow researchers to map common and distinct perturbations across pharmacological, experiential, and autonomic domains, while shared outcome measures (e.g., cognitive flexibility, affective processing, subjective experience, autonomic regulation, and biological markers of plasticity or inflammation) would facilitate cross-modal comparisons. Longitudinal designs could further clarify how acute changes in neurochemical, experiential, or autonomic domains translate into durable psychological outcomes. Together, these methodological advances will enable a more rigorous characterization of how serotonergic, experiential, and autonomic pathways interact to produce therapeutic change and may ultimately support principled sequencing strategies.

As an extension of the pharmacological pathway in this framework, selective pharmacological probes enable causal dissection of serotonergic, glutamatergic, and plasticity-related mechanisms underlying full-dose psychedelic states. Selective 5-HT2A antagonists such as ketanserin reliably block the subjective and neural effects of psilocybin and LSD in humans, providing a powerful tool for isolating 5-HT2A-dependent components of the acute response (Quednow et al., 2012; Holze et al., 2021a). Downstream plasticity mechanisms may be probed using agents that modulate mTOR or BDNF-related pathways; although rapamycin has been used in human neuroplasticity research (Abdallah et al., 2020), its application in psychedelic studies remains limited, and even single-dose administration warrants careful evaluation due to immunostimulant and -suppressive, and metabolic effects (Lamming et al., 2012; Mannick et al., 2018; O'Shea et al., 2022) and the potential to interfere with 5-HT2A-dependent mTOR-mediated plasticity (Ly et al., 2018; Vargas et al., 2023). Glutamatergic contributions can be examined using NMDA-receptor modulators such as ketamine and memantine (Krystal et al., 1994; Parsons et al., 2008), and a sequential, low-dose ketamine-psychedelic design would allow dissociation of glutamatergic and serotonergic contributions to human plasticity. Despite their complementary mechanisms, ketamine-psychedelic combination studies have not yet been conducted in controlled human settings, leaving open the opportunity to use these agents as orthogonal probes of NMDA- versus 5-HT2A-dependent plasticity. Partial NMDA agonists such as D-cycloserine may further help dissociate learning-related plasticity from 5-HT2A-mediated effects (Norberg et al., 2008). Together, these probes offer a causal route to linking receptor-level and glutamatergic plasticity mechanisms with downstream interoceptive-autonomic processes within the three-pathway framework.

## Conclusion

Single low-dose psychedelics, high-ventilation breathwork, and VNS each perturb different components of the systems engaged during full-dose psychedelic therapy, offering a set of complementary mechanistic probes. Low-dose psychedelics isolate 5-HT2A-mediated neurochemical and plasticity-related effects with minimal experiential or autonomic confounds; breathwork elicits intense experiential and autonomic shifts without serotonergic pharmacology; and VNS selectively modulates vagal-autonomic and interoceptive pathways. Examining these approaches within shared experimental frameworks enables causal comparisons across neurochemical, experiential, and autonomic mechanisms, clarifying how these domains interact to produce therapeutic change and informing the development of safer, more scalable, and more precisely targeted interventions.

## Data Availability

The original contributions presented in the study are included in the article/supplementary material, further inquiries can be directed to the corresponding author.
